# A software tool for creating simulated outbreaks to benchmark surveillance systems

**DOI:** 10.1186/1472-6947-5-22

**Published:** 2005-07-14

**Authors:** Christopher A Cassa, Karin Iancu, Karen L Olson, Kenneth D Mandl

**Affiliations:** 1 Children's Hospital Informatics Program, Informatics Program – Mandl Group, 1 Autumn St, #721, Children's Hospital Boston, Boston, MA 02115, USA; 2 Clinical Decision Making Group, Computer Science and Artificial Intelligence Laboratory, Massachusetts Institute of Technology, Cambridge, MA 02139, USA; 3 Harvard Medical School, Boston, MA 02115, USA; 4 Harvard/MIT Division of Health Sciences and Technology, Cambridge, MA 02139, USA

## Abstract

**Background:**

Evaluating surveillance systems for the early detection of bioterrorism is particularly challenging when systems are designed to detect events for which there are few or no historical examples. One approach to benchmarking outbreak detection performance is to create semi-synthetic datasets containing authentic baseline patient data (noise) and injected artificial patient clusters, as signal.

**Methods:**

We describe a software tool, the AEGIS Cluster Creation Tool (AEGIS-CCT), that enables users to create simulated clusters with controlled feature sets, varying the desired cluster radius, density, distance, relative location from a reference point, and temporal epidemiological growth pattern. AEGIS-CCT does not require the use of an external geographical information system program for cluster creation. The cluster creation tool is an open source program, implemented in Java and is freely available under the Lesser GNU Public License at its Sourceforge website. Cluster data are written to files or can be appended to existing files so that the resulting file will include both existing baseline and artificially added cases. Multiple cluster file creation is an automated process in which multiple cluster files are created by varying a single parameter within a user-specified range. To evaluate the output of this software tool, sets of test clusters were created and graphically rendered.

**Results:**

Based on user-specified parameters describing the location, properties, and temporal pattern of simulated clusters, AEGIS-CCT created clusters accurately and uniformly.

**Conclusion:**

AEGIS-CCT enables the ready creation of datasets for benchmarking outbreak detection systems. It may be useful for automating the testing and validation of spatial and temporal cluster detection algorithms.

## Background

The public health information infrastructure is yielding real-time access to health data, enabling new approaches to surveillance for infectious outbreaks. Prior to the laboratory confirmation or physician diagnosis of an infectious disease, ill persons may exhibit behavioral patterns, symptoms, signs, or laboratory findings that can be tracked through a variety of data sources. The process of monitoring these data is often referred to as syndromic surveillance [1-3].

Real time outbreak detection algorithms tend to focus on the temporal and spatial patterns of cases. Some detection routines, such as Cusum [4], look at just one type of pattern, while others, such as the Space-Time Scan Statistic [5] incorporate both. What these algorithms have in common is an underlying model of typical, or baseline patterns, and the goal of detection is to recognize perturbations from the baseline [1]. The outbreak detection performance of a surveillance system can be measured in terms of its ability to distill "signal" (a cluster of cases in time and/or space) from noisy baseline.

Benchmarking the performance of detection algorithms requires training and validation data. When real data are not available, simulated data are often used [6,7]. Simulated outbreaks must reflect the diversity of threats that a surveillance system is expected to encounter and detect, whether these outbreaks occur naturally or are man-made.

Our approach to validating detection algorithms is to use semi-synthetic data, that is, authentic baseline data injected with artificial signals [8]. These signals are defined by a controlled feature set of variable parameters such as the size, location, shape, and duration of simulated outbreaks. Here we describe a software tool, the AEGIS Cluster Creation Tool (AEGIS-CCT), that enables users to create simulated clusters with controlled feature sets, varying the desired cluster radius, density, distance, and relative location away from a central point. AEGIS-CCT does not require the use of an external geographic information system (GIS) program for cluster creation.

## Implementation

### Functional specifications

AEGIS-CCT can create single patient clusters as well as sets of patient clusters based on a simple geographical model. Cluster data points are outputted as comma separated variable (CSV) files, and can optionally be appended to an existing file, supplied by the user. The tool can also generate different sets of clusters that range in value over a single parameter to rigorously validate detection algorithms. To create individual clusters, the user can vary a number of relevant outbreak parameters (Table 1). AEGIS-CCT creates at least two output files each time it is executed, with file names specified by the user. One is a cluster data file that contains the artificial cluster data, and the other is a record file describing the session cluster parameters. The data file contains a cluster point identification number (assigned numerically from 0 to the number of points minus 1), the latitude and longitude of the cluster point, and the relative date of the cluster point. When generating a series of *n *clusters, the program automatically generates *n *files with appended identifiers, each as separate data files.

**Table 1:** Parameters that can be altered when creating a single cluster.

**Parameter**	**Description**
*Cluster ID Number*	User specified reference or identification number for each cluster
*Number of Points in the cluster*	Number of patients or points in the generated cluster.
*"Reference Point" GIS Location*	The latitude-longitude coordinates of a reference point, which could be a hospital or a primary care facility, for example.
*Maximum cluster radius*	The distance between the outermost point in the cluster and the center of the cluster.
*"Angle" from the reference point*	The angle of the cluster measured counter-clockwise from due east of the reference point as zero degrees, using unit circle convention.
*Distance from the reference point*	The distance between the center point of the cluster and the reference point.
*Numbers of Days the Cluster should span*	The number of days from when the first person shows symptoms to when the last person does.
*Date Algorithm*	This specifies which of the three models of temporal progression to use. Additional models can be incorporated into the software.
*Description and output filenames*	The user can specify where the cluster data and user-specified cluster description will be written.

The AEGIS-CCT is a Java package including a geospatial engine and a user interface created using the Swing toolkit. The source for the entire package is provided under the Lesser GNU Public License [9] on a sourceforge.net development site [10]. Full details and updates to AEGIS-CCT can be obtained online [11].

### Geocoding and precision of location

Programmatic methods were implemented to assign latitude-longitude coordinates to simulated cluster points, taking into account physical earth surface distances and not relying on external GIS software. Inside an AEGIS-CCT GIS class, there are three primary methods to handle these conversions. The first is a method to find the distance between two locations, which uses the specific latitude-longitude of the reference point to create a ratio of degrees per meter for north-south latitude and east-west longitude. Artificial data points are created 0.05 degrees to the north or east, and the corresponding physical distances (x, y) are calculated using the Haversine Formula, described below. The ratio is then computed, dividing the artificial data point distance by the calculated physical distance in meters on the Earth's surface. A second method finds a point that is a specific physical distance, measured at a specified angle, from a reference point. The angle is measured from the Euclidian x-axis and increased in a counter-clockwise form. The output is a second GIS data point related to the reference point by the angle and distance specified by the user. The third method finds the number of degrees of latitude and longitude per unit of physical distance in each respective direction.

### GIS precision

Placing patients on a map requires consideration of earth curvature and precise latitudes and longitudes. Spherical equations break down significantly at small distances, but the Haversine formula [12] provides computationally exact results in almost all circumstances. For this calculation, Earth has radius R, and the locations of two points in spherical coordinates (latitude and longitude) have names [lon1, lat1] and [lon2, lat2]. The Haversine Formula is calculated using the following code:

dlon = lon2 - lon1;

dlat = lat2 - lat1;

a = (sin(dlat/2)) ^2 + cos(lat1) * cos(lat2) * (sin(dlon/2)) ^2;

c = 2 * atan2(sqrt(a), sqrt(1-a));

d = R * c;

This implementation was quality tested for accuracy using a series of latitude-longitude pairs from a sample dataset, measuring distances between two data points. Those results were identical with distances calculated by commercially-available GIS software.

### Geotemporal progression

Outbreaks vary in their temporal progressions or epidemic curves. Three such progressions were implemented in AEGIS-CCT as date algorithms to model the ways in which a disease might manifest in a population over time: a random, a linear, and an exponential growth spread. Additional epidemiological date algorithms can be added by other users to the AEGIS-CCT by implementing a method to assign a specific temporal distribution within an array in Java.

**Figure 1:**
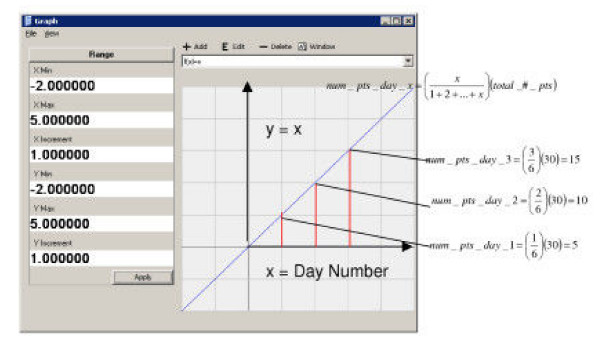
An example of the linear date algorithm estimation for thirty points spanning three days. The x-axis represents the day number.

**Figure 2:**
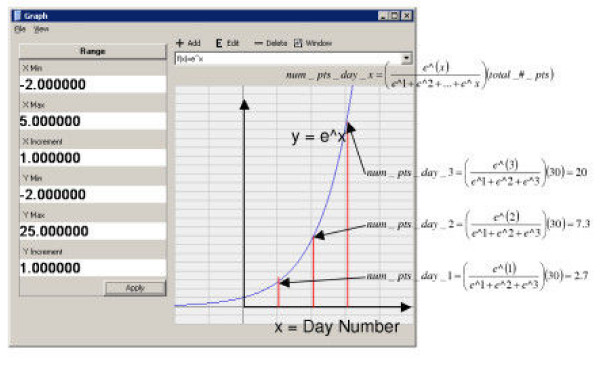
An example of the exponential date algorithm estimation for thirty points spanning three days. The x-axis represents the day number.

For the random algorithm, a random number is generated that falls within the range of the number of days in the cluster, producing a random distribution. For the linear distribution, the day value is divided by the total day values and multiplied by the total number of points to determine the fraction of the total points to be injected each day. Scaling by a multiplier can alter the rate of linear growth. Similarly for the exponential distribution, the numerical value of *e*^(multiplier * day number) ^is divided by the sum of *e*^(multiplier * day number)^, for all day values. This ratio is then multiplied by the total number of points to determine the number of points that occur each day. Examples of the linear and exponential growth modified probability distribution estimations for date distribution are presented in Figures 1 and 2.

## Results

### Analysis of accuracy and uniformity of patient cluster data

Semi-synthetic datasets created by the cluster generator fall within a specified set of parameter-based boundaries. Cluster data points are created randomly within the domain defined by those parameters, so it is important to verify that the clusters are accurately created and are close to uniformly generated.

To measure the uniformity of generated clusters, 10 test clusters were created with 100 points in each cluster. The centroid of each set of cluster points was then calculated and compared to the specified center point of that cluster. In every case, the cluster centroid was within five percent of the specified cluster radius, in distance, from the specified center point. This result demonstrates that the datasets are uniform, within a small threshold, when they contain a sufficient number of points, as would be expected with a random distribution.

To measure the accuracy of the geocoding engine, 360 clusters were made, forming a circle, around a single center point, varying the angle evenly (one degree added per cluster,) and they each had a cluster center point that fell precisely along the circle defined by all points at the same radial distance from the original counterpoint. This same test was conducted at five randomly selected latitude-longitude locations and the same results were obtained.

### Sample cluster parameters and output

Two sample scenarios are described below, and their parameters are listed in Table 2. The first example demonstrates the creation of a single artificial cluster and the second example demonstrates the creation of several clusters at various angles around a single reference point. The first cluster includes cases in a simulated outbreak spanning five days.

**Table 2:** Example of single cluster parameters and multiple cluster parameters.

**Parameter**	**Type of Cluster**	
	**Single Cluster**	**Set of Clusters Varying Angle**
Cluster ID	100	101 [1:4]
Number of Points in Cluster	30	30
Reference Point Latitude	42.35666	42.35666
Reference Point Longitude	-71.09516	-71.09516
Cluster Radius	600 m	400 m
Angle from reference point	90	[varies, see below]
Distance from reference point	1600 m	3000 m
Number of Days	5	5
Time-growth Pattern	Linear	Linear
Cluster Description	Linear time-growth cluster north of center point	Varied angle around center point and created 4 clusters.
Number of Clusters	N/A	4
Minimum Angle	N/A	0
Maximum Angle	N/A	270

The single linear time-growth cluster was placed approximately 1600 m due north of a center point at longitude -71.09516 and latitude 42.35666. AEGIS-CCT outputted a CSV file and partial results are listed in Table 3. There are two points on the first day, four on the second day, six on the third day, linearly increasing to include thirty patient points by day 5.

**Table 3:** Sample output to a comma separated value file from AEGIS-CCT. Note: Values are point identification number, longitude, latitude and day number.

0,-71.09600452536358,42.37455407329273,1
1,-71.10149672138236,42.365894560466806,1
2,-71.0954755413253,42.373890954435524,2
3,-71.08968377859539,42.37242100053542,2
4,-71.09281946336338,42.36955324904336,2
5,-71.09564524977307,42.371694560897,2
6,-71.09345472615571,42.370979504450304,3
7,-71.09983295495935,42.369683605959985,3
8,-71.09781606117451,42.37282397457113,3
9,-71.09685871099056,42.37540065852763,3
10,-71.0921214185705,42.37216701505921,3
... (to point with ClusterID 29)

**Figure 3:**
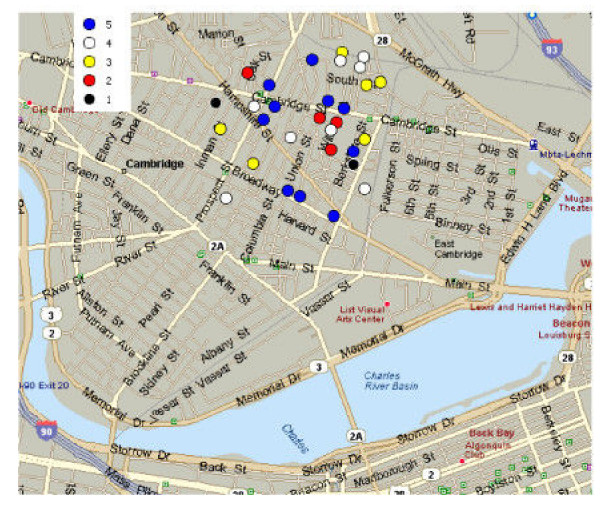
A single linear time-growth cluster north of center point.

**Figure 4:**
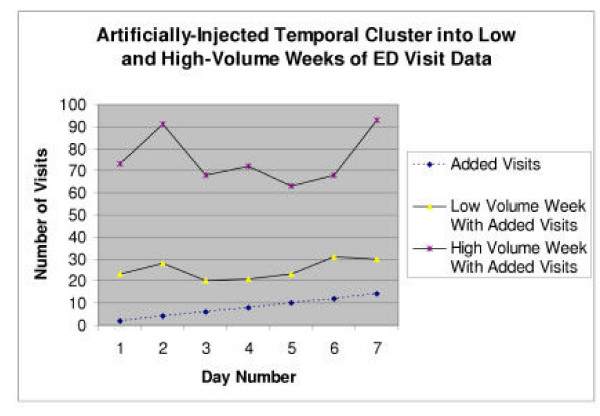
Artificially-Injected Temporal Cluster into Low and High-Volume Weeks of Children's Hospital Boston ED Visit Data: An artificially-generated cluster (dashed line at bottom) containing a total of 56 additional points, linearly increasing in magnitude over a 7 day span, was added to two separate weeks of Children's Hospital Boston temporal visit data. The first week of data was from a low-volume week, containing a total of 120 authentic patient visits with an additional 56 artificially-generated visits while the second series contains a total of 472 authentic visits with the same 56 artificially-generated visits appended. While growth in the low-volume week is visible by inspection, it is difficult to notice the artificially added visits in a higher-volume week.

The output CSV can be imported into a GIS analysis tool. A map made from AEGIS-CCT output using MapPoint 2002 (Microsoft Corporation, Redmond, WA) is presented in Figure 3. The temporal progression (linear-growth algorithm) is indicated by the shaded color of coordinate points for each simulated case as shown in the legend. The temporal growth pattern of a similar injected linear-growth cluster spanning 7 days (containing a total of 56 points) is graphed when appended to a low-volume week (120 visits, 07/15-21/2001) and a high-volume week (472 visits, 01/14-20/2001) in Figure 4.

**Figure 5:**
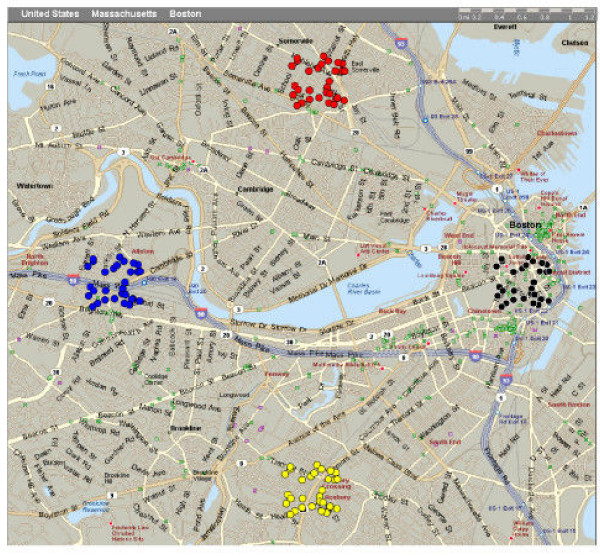
Creation of a series of four clusters around the center point (with the angle varied.)

In the second example, the angle around the center point was varied, creating a series of four clusters. The series cluster generator automatically created four files, and each file was imported into MapPoint, and charted in a different color, as shown in Figure 5.

The CCT can create new data files or it can append cluster data to an existing file. The artificial portion of a semi-synthetic dataset can then be automatically combined with baseline patient distributions by specifying a file that contains the baseline data. In future versions, the CCT may provide an xml schema to which input baseline data files should conform.

### Limitations

AEGIS-CCT does not yet have built-in procedures to generate more complicated time-series or spatial distributions. Extensibility was taken very seriously when creating AEGIS-CCT, and sufficient abstractions were made to allow for ease of adding additional models. The generator is also extensible in other ways so that additional parameters can be added if they are easily computable. Parameters can be added or deleted by updating the GUI and modifying the GenerateCluster method in the main geospatial class. Potential areas for expansion in future versions include log-linear and logarithmic time-series models as well as Gaussian spatial distributions. It will be necessary to determine what the most pertinent and physically realistic models of syndromic spread are. Once the most realistic scenarios are assessed and modeled, they can be programmatically implemented and incorporated into the cluster creation tool. As long as these distributions can be implemented using Java methods, it is possible to quickly add another temporal distribution to AEGIS-CCT.

## Conclusion

Evaluation of surveillance systems for the early detection of outbreaks is particularly challenging [13] when the systems are designed to detect events for which there are a few or no historical examples. Some real-time surveillance systems are designed to provide early warning of a biological attack. Fortunately, few people have been infected with biological warfare agents, although there are notable exceptions. For example, residents of Sverdlovsk were exposed in 1979 during an accidental release of anthrax from a weapons plant [14] and there were eleven infections, resulting in five deaths in the Florida, New York and Washington DC mailed-anthrax attacks in 2001 [15]. In the absence of sufficient real outbreak data, measuring the detection performance of a system requires simulation. AEGIS-CCT enables the ready creation of datasets for benchmarking outbreak detection systems.

## Availability and requirements

### Lists the following

* Project name: AEGIS Cluster Creation Tool

* Project home page: 

* Operating system(s): Platform independent

* Programming language: Java

* Other requirements: Java 1.3.1 or higher

* License: e.g. GNU LGPL

* Any restrictions to use by non-academics: none

## List of abbreviations

GIS – Geographical Information Systems

GUI – Graphical User Interface

CSV – Comma-Separated Values

## Competing interests

The author(s) declare that they have no competing interests.

## Authors' contributions

CC implemented the GIS engine and dataset management, thoroughly tested of program, and was the primary author of the manuscript. KI helped implement the graphical user interface and the file read and write components. KO provided datasets and domain knowledge of specific issues relevant to the evaluation of detection performance. KM conceived of the study and participated in its design and coordination. All authors read and approved the final manuscript.

## Pre-publication history

The pre-publication history for this paper can be accessed here:


